# Proteasome activity is required for the initiation of precancerous pancreatic lesions

**DOI:** 10.1038/srep27044

**Published:** 2016-05-31

**Authors:** Takaki Furuyama, Shinji Tanaka, Shu Shimada, Yoshimitsu Akiyama, Satoshi Matsumura, Yusuke Mitsunori, Arihiro Aihara, Daisuke Ban, Takanori Ochiai, Atsushi Kudo, Hiroshi Fukamachi, Shigeki Arii, Yoshiya Kawaguchi, Minoru Tanabe

**Affiliations:** 1 Department of Molecular Oncology, Graduate School of Medicine, Tokyo Medical and Dental University, Tokyo, Japan; 2 Department of Hepato-Biliary-Pancreatic Surgery, Graduate School of Medicine, Tokyo Medical and Dental University, Tokyo, Japan; 3 Department of Clinical Application, Center for iPS cell Research and Application (CiRA), Kyoto University, Kyoto, Japan

## Abstract

Proteasome activity is significantly increased in advanced cancers, but its role in cancer initiation is not clear, due to difficulties in monitoring this process *in vivo*. We established a line of transgenic mice that carried the ZsGreen-degron^ODC^ (Gdeg) proteasome reporter to monitor the proteasome activity. In combination with *Pdx-1-Cre;LSL-Kras*^*G12D*^ model, proteasome activity was investigated in the initiation of precancerous pancreatic lesions (PanINs). Normal pancreatic acini in *Gdeg* mice had low proteasome activity. By contrast, proteasome activity was increased in the PanIN lesions that developed in *Gdeg;Pdx-1-Cre;LSL-Kras*^*G12D*^ mice. Caerulein administration to *Gdeg;Pdx-1-Cre;LSL-Kras*^*G12D*^ mice induced constitutive elevation of proteasome activity in pancreatic tissues and accelerated PanIN formation. The proteasome inhibitor markedly reduced PanIN formation in *Gdeg;Pdx-1-Cre;LSL-Kras*^*G12D*^ mice (*P* = 0.001), whereas it had no effect on PanIN lesions that had already formed. These observations indicated the significance of proteasome activity in the initiation of PanIN but not the maintenance *per se*. In addition, the expressions of pERK and its downstream factors including cyclin D1, NF-κB, and Cox2 were decreased after proteasome inhibition in PanINs. Our studies showed activation of proteasome is required specifically for the initiation of PanIN. The roles of proteasome in the early stages of pancreatic carcinogenesis warrant further investigation.

Pancreatic cancer is one of the most deadly human cancers. It is usually diagnosed at an advanced stage and is resistant to therapy^1^. Therefore, there is a dire need for new therapeutic approaches based on a better understanding of the biology of this disease^2^. Pancreatic ductal adenocarcinoma (PDAC), the most common form of pancreatic cancer, arises from a spectrum of preneoplastic mucinous lesions with ductal morphology, namely pancreatic intraepithelial neoplasias (PanINs)^3^. The earliest detectable mutations found in PanINs are activating mutations of the *KRAS* gene. More than 90% of cases of PanINs of all grades have *KRAS* mutations^4^. In the mouse model, constitutive expression of mutant KRAS in pancreatic acinar cells spontaneously induce PanINs^5^. PanIN formation is preceded by acinar-to-ductal metaplasia (ADM), a process of dedifferentiation of acinar cells to ductal cells^6^. Acute pancreatitis induces transient ADM followed by reversion to normal acinar cell morphology in the absence of mutant KRAS^7^. By contrast, mutant KRAS dramatically accelerates the ADM to PanIN sequence^8^. These observations indicate the significance of *KRAS* mutations for the initiation of PanIN, but the detailed mechanisms remain unknown.

*RAS* mutant cancers show selective dependencies on activities that are not regulated by Ras, but that are needed to adapt to the stress induced by the process of transformation. This phenomenon is referred to as “non-oncogene addiction”^9^. One of the key components of such activities is the proteasome. The proteasome degrades short-lived regulatory proteins involved in various cellular functions. Additionally, it removes misfolded and damaged proteins^10^. In cancer cells, proteotoxic stress is a frequent occurrence resulting from excessive amounts of misfolded proteins that lead to enhanced proteasome activity^11^. Cancer cells with mutant *KRAS* show selective addiction to proteasome activity, and proteasome inhibition shows synthetic lethality with mutant *KRAS*^12^,^13^.

Increased protein turnover is one aspect of advanced cancers including PDAC, but a recent study suggests that it begins much earlier than previously appreciated and can be an early event in development of PDAC^14^. Thus, the proteasome potentially has roles in the early stages of cancer initiation. But so far, its impact on cancer initiation has not been elucidated. One of the reasons is the difficulty in evaluating proteasome activity *in vivo*. Dantuma *et al.* developed Ub^G76V^-GFP proteasome reporter system that allows rapid quantification of ubiquitin-proteasome-dependent proteolysis in living cell^15^. They further developed a transgenic mouse model with this reporter for *in vivo* analysis of the ubiquitin/proteasome system^16^. In addition, Liu *et al.* reported another transgenic mouse model with a reporter system of GFP modified by carboxyl fusion of a consensus ubiquitination signal sequence (CL1) isolated from yeast^17^. These reporter systems can monitor *in vivo* proteasome activity, and also reflect the ubiquitination in living cells. Proteasomal degradation of most proteins depends on their ubiquitination, whereas the degron sequence of ornithine decarboxylase (ODC) is known to be directly recognized by the proteasome enzyme without ubiquitination^18^. Vlashi *et al.* and our group previously evaluated the ubiquitin-independent proteasome activity in living cells using ZsGreen-degron^ODC^ (Gdeg) reporter^19^,^20^,^21^. Gdeg reporter system consists of the green fluorescent protein ZsGreen fused to the degron of ODC, whose accumulation can be reflected by the ubiquitin-independent proteasome activity. Though Gdeg reporter has also been applied to *in vivo* studies such as tumor xenograft models^22^, a transgenic mouse model with this ZsGreen-degronODC reporter system has not been reported to date.

In the present study, we developed a transgenic mouse model with this reporter system, which enabled us to monitor the proteasome activity. Using this mouse model, we revealed constitutive elevation of proteasome activity during PanIN formation. Additionally, proteasome inhibition markedly reduced PanIN formation with decreased expressions of pERK and its downstream factors including cyclin D1, nuclear factor-kappa B (NF-κB), and Cox2. Our data show crucial roles of proteasome activity in the initiation of PanIN.

## Results

### Generation of *Gdeg* transgenic mice

**Figure 1:**
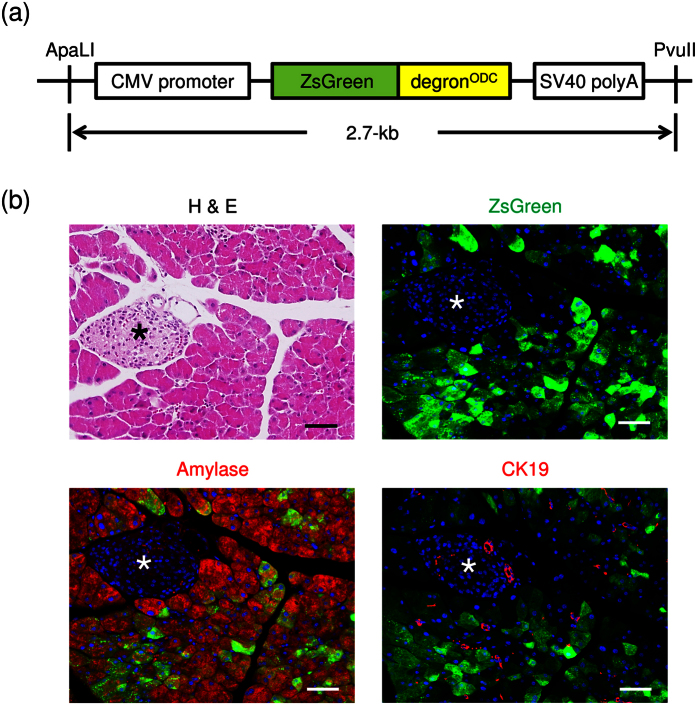
Generation of *Gdeg* transgenic mice. (**a**) Structure of the Gdeg transgene. (**b**) H&E staining, fluorescence imaging, and immunofluorescence staining for amylase and cytokeratin19 (CK19) of the pancreas of *Gdeg* mice. Asterisks indicate islet cells. Bar, 100 μm.

In order to monitor the proteasome activity, transgenic mice with the Gdeg reporter were constructed (referred to as *Gdeg* mice). The transgene consisted of the Gdeg reporter ligated between the cytomegalovirus (CMV) promoter and the simian virus 40 (SV40) poly-adenylation site (Fig. 1a). We first performed primary cell culture to confirm the expression of the transgene. Primary cultured fibroblasts exhibited no accumulation of Gdeg under normal conditions, but all of them responded to treatment with the proteasome inhibitors MG132 and bortezomib and showed pronounced accumulation of Gdeg (Supplementary Fig. S1). This observation indicates constitutive expression of the transgene in *Gdeg* mice. Histologically, abundant accumulation of Gdeg was observed in the pancreas (Fig. 1b). Immunohistochemical analysis revealed that the majority of amylase positive acinar cells exhibited accumulation of Gdeg, whereas no accumulation of Gdeg was found in ductal cells expressing cytokeratin 19 (CK19) or islet cells (Fig. 1b). Expression of *Gdeg* mRNA in other cell types showing no Gdeg accumulation was confirmed by RT-PCR analysis using laser capture microdissection (LCM) and fluorescence-activated cell sorting (FACS) assays (Supplementary Fig. S2). These findings suggest that proteasome activity is relatively low in normal pancreatic acini. No pathological findings due to gene transfection were observed in the pancreas or other tissues.

### Transient elevation of proteasome activity following caerulein stimulation

**Figure 2:**
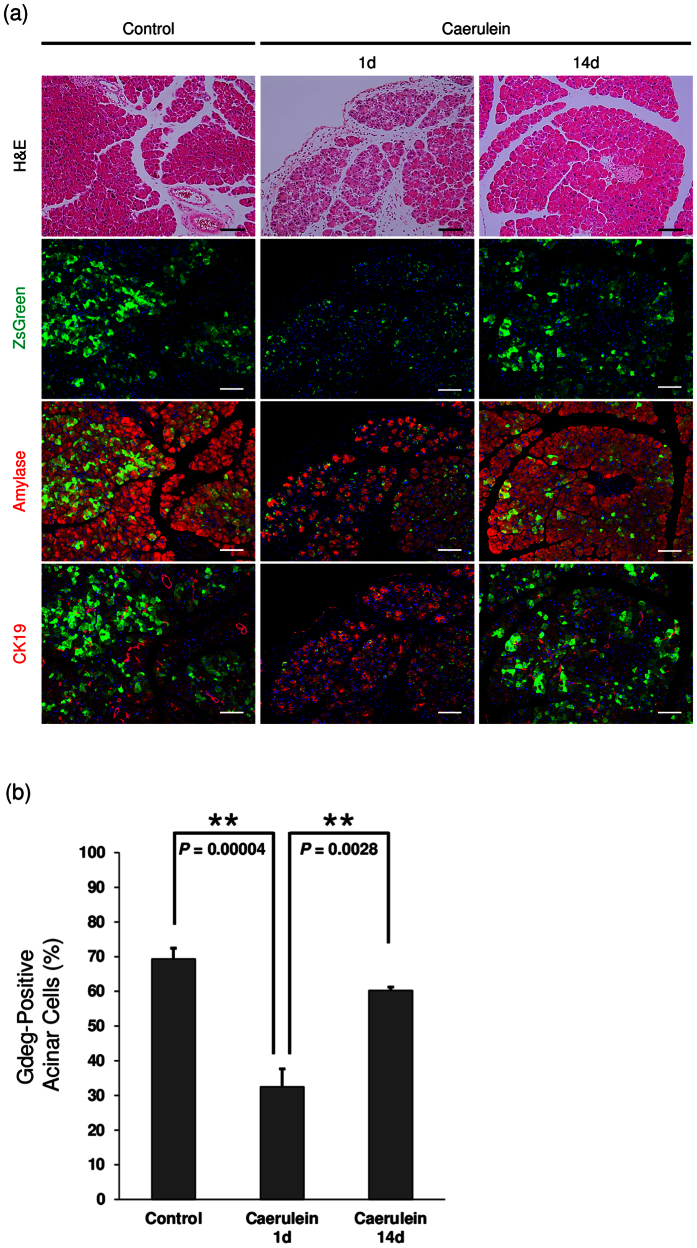
Transient elevation of proteasome activity following caerulein stimulation. (**a**) H&E staining, Alcian blue staining, fluorescence imaging, and immunofluorescence staining for amylase and cytokeratin19 (CK19) of the pancreas of caerulein-treated *Gdeg* mice (n = 4). Bar, 100 μm. (**b**) The Gdeg-positive rate in acinar cells was quantified in each group. Values are shown as mean ± SD. The Gdeg-positive rates were 69.3% ± 3.1% in control mice, 32.5% ± 5.2% in mice at day 1, and 60.2% ± 1.0% in mice at day14, respectively.

Previous studies have shown that caerulein stimulation accelerates *Kras*^*G12D*^-mediated PanIN formation^8^. To evaluate the effect of caerulein stimulation on proteasome activity, *Gdeg* mice were administered with caerulein and the change of proteasome activity was analyzed. As previously reported, caerulein induced transient pancreatic inflammation and ADM, which were replaced by normal acini within 2 weeks. Gdeg accumulation in acinar cells transiently decreased after caerulein administration (day1), but recovered within 2 weeks (Fig. 2a). This transient Gdeg reduction was improved by proteasome inhibitor MG132 without affecting the pancreatic damage (Supplementary Fig. S3), indicating potentially transient elevation of proteasome activity in the pancreas after caerulein stimulation. As shown in Fig. 2b, the Gdeg-positive rate in acinar cells was significantly decreased in mice at day1, compared to the control and mice at day14 after caerulein administration (*P* = 0.00004 and *P* = 0.0028, respectively).

### Constitutive elevation of proteasome activity in *Kras* ^*G12D*^-mediated PanIN formation

**Figure 3:**
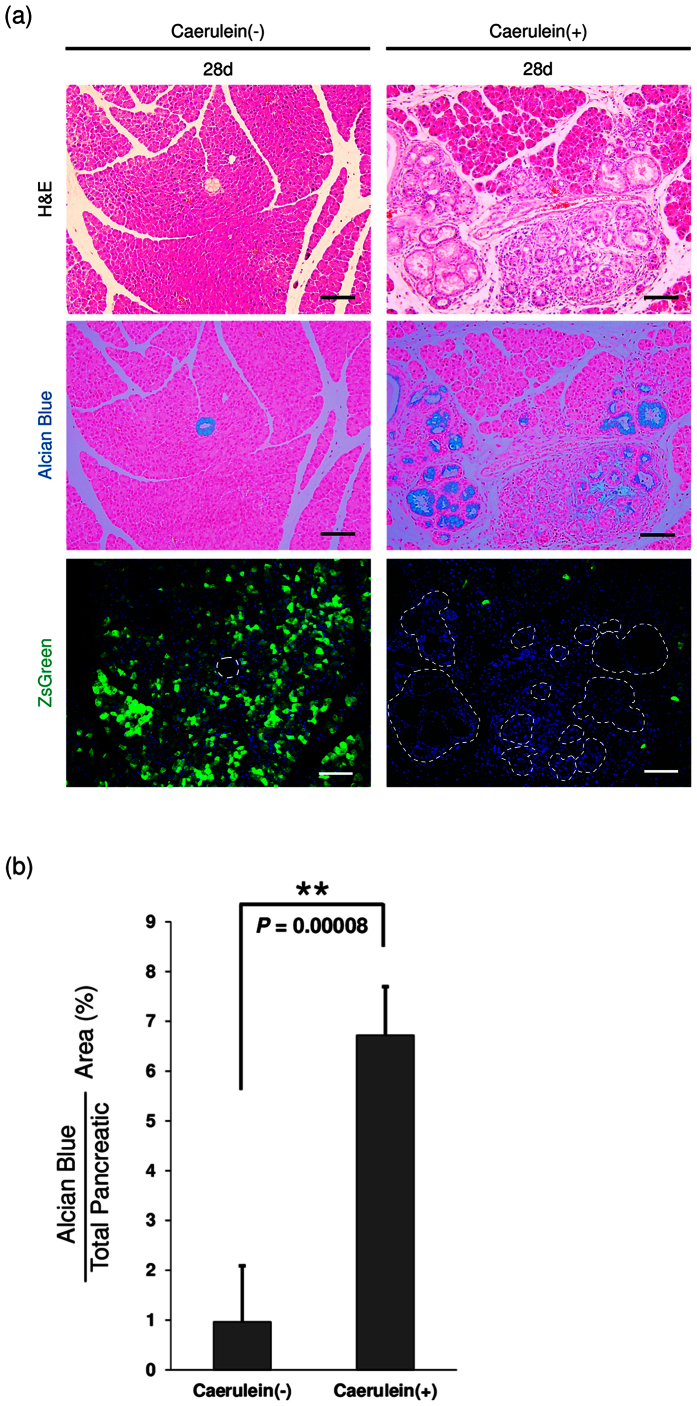
Visualization of proteasome activity in *Kras*^*G12D*^-mediated PanIN lesions. (**a**) H&E staining, Alcian blue staining, and fluorescence imaging of the pancreas of *Gdeg;Pdx-1-Cre*;*LSL-Kras*^*G12D*^ mice with or without caerulein treatment. Mice were intraperitoneally injected with caerulein or PBS, and analyzed on day 28 (n = 4). Dashed lines on the fluorescence images mark PanIN lesions. Bar, 100 μm. (**b**) Quantification of Alcian blue positive areas in each group. Values are shown as mean ± SD.

To monitor proteasome activity during PanIN formation, our *Gdeg* mice were crossed to the well-established *Pdx-1-Cre; LSL-Kras*^*G12D*^ model^5^ to generate *Gdeg;Pdx-1-Cre;LSL-Kras*^*G12D*^ triple transgenic mice. The morphology of the pancreatic tissues of *Gdeg;Pdx-1-Cre*;*LSL-Kras*^*G12D*^ mice was almost normal and formation of Alcian blue-positive PanINs was rare (Fig. 3a, upper and middle left). These results are consistent with previous observations in *Pdx-1-Cre;LSL-Kras*^*G12D*^ model. Intense accumulation of Gdeg was observed in pancreatic acini, which was similar to normal pancreatic tissue in *Gdeg* mice. In contrast, accumulation of Gdeg completely vanished in PanIN lesions (Fig. 3a, lower left). Though PanIN lesions spontaneously develop in *Pdx-1-Cre;LSL-Kras*^*G12D*^ model, it takes long time to form high grade PanINs and the proportion of high grade PanIN lesions is limited in the pancreatic tissues^5^. For sufficient PanIN formation, additional factors such as caerulein stimulation are required in this model^8^. Thus, we next administered caerulein to triple transgenic mice. As expected, caerulein treatment markedly accelerated PanIN formation. At 28 days after caerulein treatment, the pancreatic tissues contained massive PanINs and its precursor lesions (Fig. 3a, upper and middle right). The Alcian blue positive area of the pancreas was significantly increased in mice with caerulein treatment compared to mice without caerulein treatment (6.72 ± 0.98% vs 0.96% ± 1.13%, respectively, *P* = 0.00008) (Fig. 3b). Similar to those observed in mice without caerulein stimulation, PanINs exhibited elevated proteasome activity with no Gdeg accumulation. Expression of *Gdeg* mRNA was confirmed by LCM/RT-PCR in the PanIN cells showing no Gdeg accumulation (Supplementary Fig. S4).

### Suppression of *Kras* ^*G12D*^-mediated PanIN formation under proteasome inhibition

**Figure 4:**
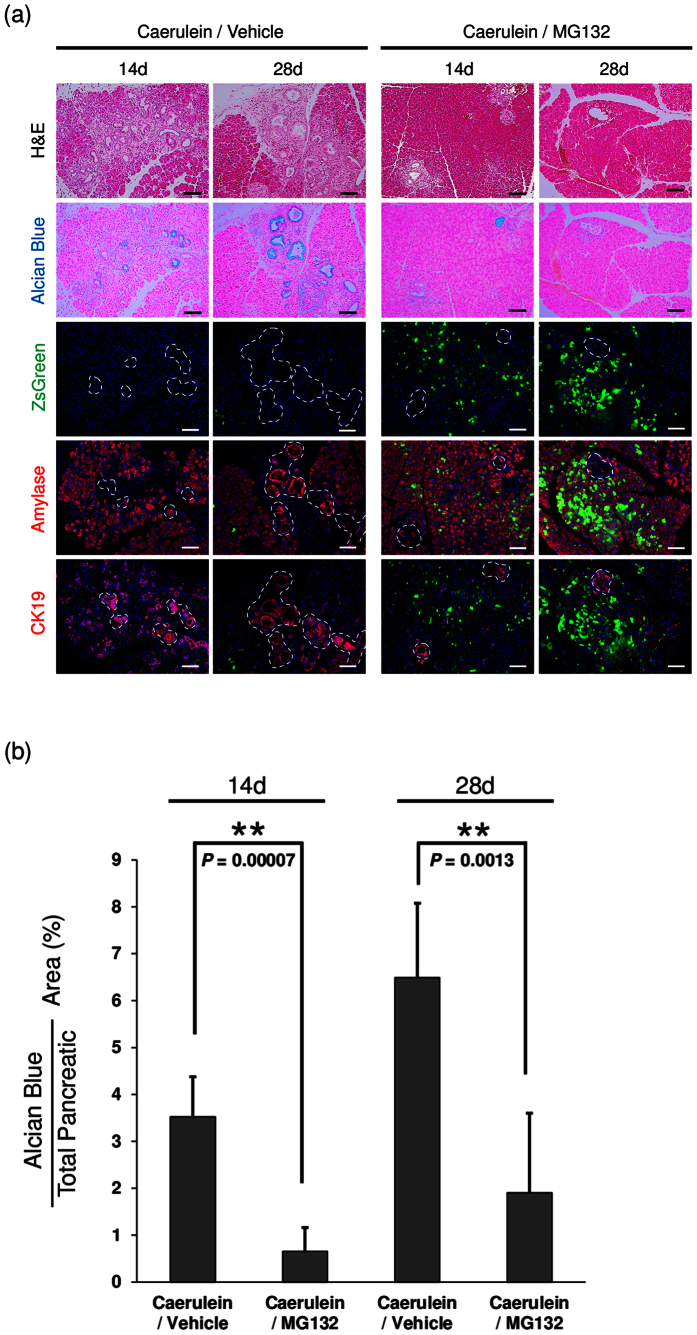
Suppression of *Kras*^*G12D*^ -mediated PanIN formation under proteasome inhibition. (**a**) H&E staining, Alcian blue staining, fluorescence imaging, and immunofluorescence staining for amylase and cytokeratin19 (CK19) of the pancreas of caerulein-treated *Gdeg;Pdx-1-Cre*;*LSL-Kras*^*G12D*^ mice with or without MG132 administration (n = 3). Dashed lines on the fluorescence images mark PanIN lesions. Bar, 100 μm. (**b**) Quantification of Alcian blue positive areas in each group. Values are shown as mean ± SD.

To address the significance of proteasome activity in the initiation of PanIN, we administered caerulein to *Gdeg;Pdx-1-Cre*;*LSL-Kras*^*G12D*^ mice in the presence or absence of MG132. Without MG132 treatment, Alcian blue-positive PanIN lesions were identified at day 14, and expanded at day 28. Elevation of proteasome activity in the pancreatic tissues persisted both at day14 and 28. Proteasome inhibition with MG132 dramatically reduced PanIN formation and maintained Gdeg accumulation in caerulein-treated *Gdeg;Pdx-1-Cre*;*LSL-Kras*^*G12D*^ mice both at day 14 and 28 (Fig. 4a). In contrast, there was no Gdeg accumulation in PanIN lesions even after MG132 treatment. Immunofluorescence analysis showed that proteasome inhibition preserved dense amylase positive acinar cells and reduced aberrant lesions expressing ductal marker CK19 (Fig. 4a), indicating suppression of PanINs. To thoroughly compare the extent of PanIN formation, we quantified the Alcian blue positive area in each case. As shown in Fig. 4b, the Alcian blue positive area of the pancreas was significantly decreased in mice with MG132 treatment compared to mice without MG132 treatment both at day 14 (0.65 ± 0.51% vs 3.52 ± 0.85%, respectively, *P* = 0.00007) and day 28 (1.90 ± 1.70% vs 6.49% ± 1.58%, respectively, *P* = 0.0013). These data underscore the requirement of proteasome activity for the initiation of PanIN.

### Expression of Ras signaling-related proteins in PanIN lesions under proteasome inhibition

**Figure 5:**
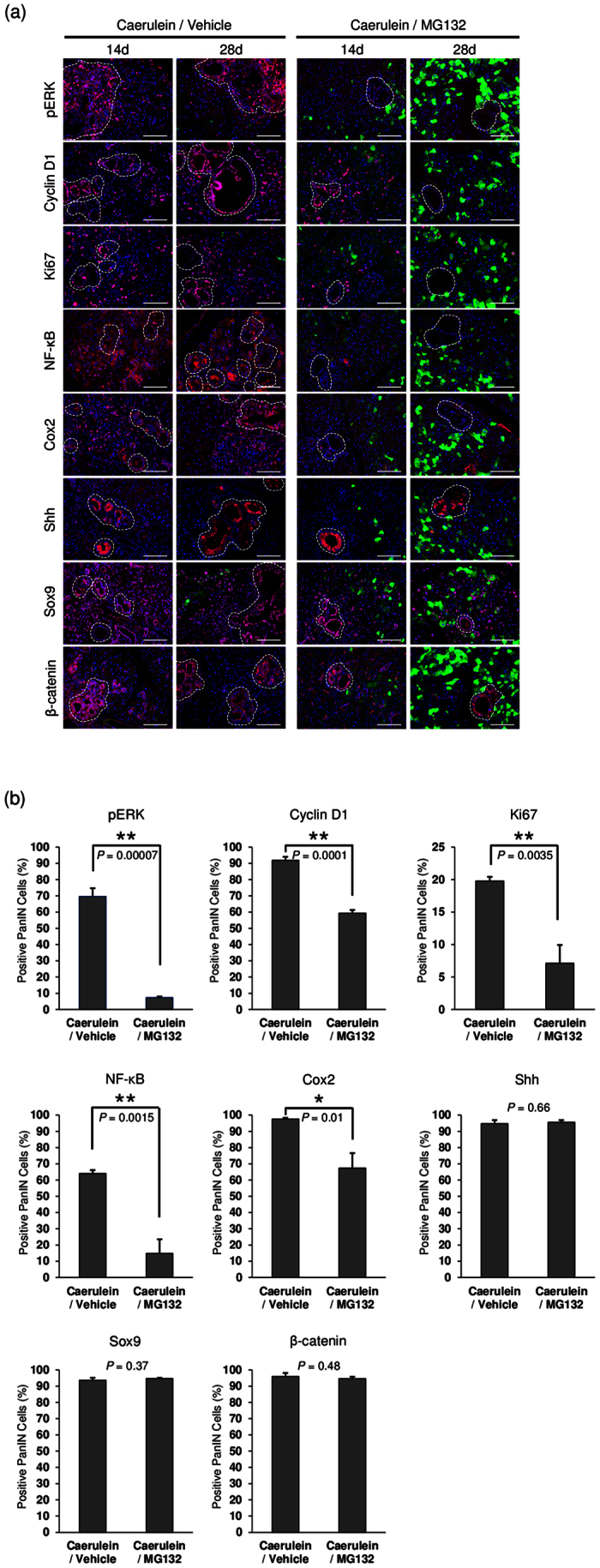
Expression of Ras signaling-related proteins in PanIN lesions under proteasome inhibition. (**a**) Immunofluorescence staining for pERK, cyclin D1, Ki67, NF-κB, Cox2, Shh, Sox9, and β-catenin of the pancreas of caerulein-treated *Gdeg;Pdx-1-Cre*;*LSL-Kras*^*G12D*^ mice with or without MG132 administration (n = 3). Dashed lines on the fluorescence images mark PanIN lesions. Bar, 100 μm. (**b**) Quantification of the positive staining rates of pERK, cyclin D1, Ki67, NF-κB, Cox2, Shh, Sox9, and β-catenin at day 28. Values are shown as mean ± SD.

Ras regulates a variety of signaling cascades, among which the MAPK/ERK pathway is the most important^23^,^24^. In order to gain additional insight into the effects of proteasome inhibition during PanIN initiation, we examined the expression of pERK and its downstream factors, Cyclin D1 and NF-κB, by immunofluorescence analysis on pancreatic tissues of caerulein-treated *Gdeg;Pdx-1-Cre*;*LSL-Kras*^*G12D*^ mice with or without MG132 treatment at day 14 and 28 (Fig. 5a). The corresponding Alcian blue staining was used as a positive PanIN marker in Supplementary Fig. S5. We also quantified the positive staining rates of each factors in PanIN cells at day 28 (Fig. 5b). The MAPK/ERK pathway was active in PanIN lesions, as determined by strong nuclear and cytoplasmic pERK staining. The positive staining rate of pERK in PanIN cells was significantly decreased in mice with MG132 treatment compared to mice without MG132 treatment (7.35 ± 0.70% vs 69.6% ± 5.1%, respectively, *P* = 0.00007). As for pERK downstream factors, strong expression of cyclin D1 was observed in the nuclei of PanINs and surrounding precursor lesions. The positive staining rate of cyclin D1 was significantly decreased under proteasome inhibition (59.3% ± 1.9% with MG132 treatment vs 91.8% ± 2.3% without MG132 treatment, *P* = 0.0001). We also observed that proliferation marker Ki67 showed a similar staining pattern to cyclin D1, and Ki67-positive cells were decreased by MG132 treatment (7.1% ± 2.8% with MG132 treatment vs 19.8% ± 0.6% without MG132 treatment, *P* = 0.0035). Nuclear and cytoplasmic staining of NF-κB was evident in PanINs in mice without MG132 treatment, whereas the expression of NF-κB was rarely seen in mice with MG132 treatment. The positive staining rate of NF-κB was significantly different between two groups (14.8% ± 8.8% with MG132 treatment vs 64.0% ± 2.1% without MG132 treatment, *P* = 0.0015). *Cox2* is one of the target genes of NF-κB, and is responsible for the generation of several inflammatory mediators^25^. Similar to NF-κB, abundant Cox2-positive cells were observed in PanIN lesions, and the positive staining rate of Cox2 in PanIN cells was significantly decreased under proteasome inhibition (67.3% ± 9.2% with MG132 treatment vs 97.6% ± 0.7% without MG132 treatment, *P* = 0.01). We also investigated other oncogenic factors such as Shh^26^, Sox9^27^ and β-catenin^28^, but the positive staining rates of these factors were unaffected by proteasome inhibition (*P* = 0.66, 0.37, and 0.48, respectively).

### The effect of proteasome inhibitor after PanIN formation

**Figure 6:**
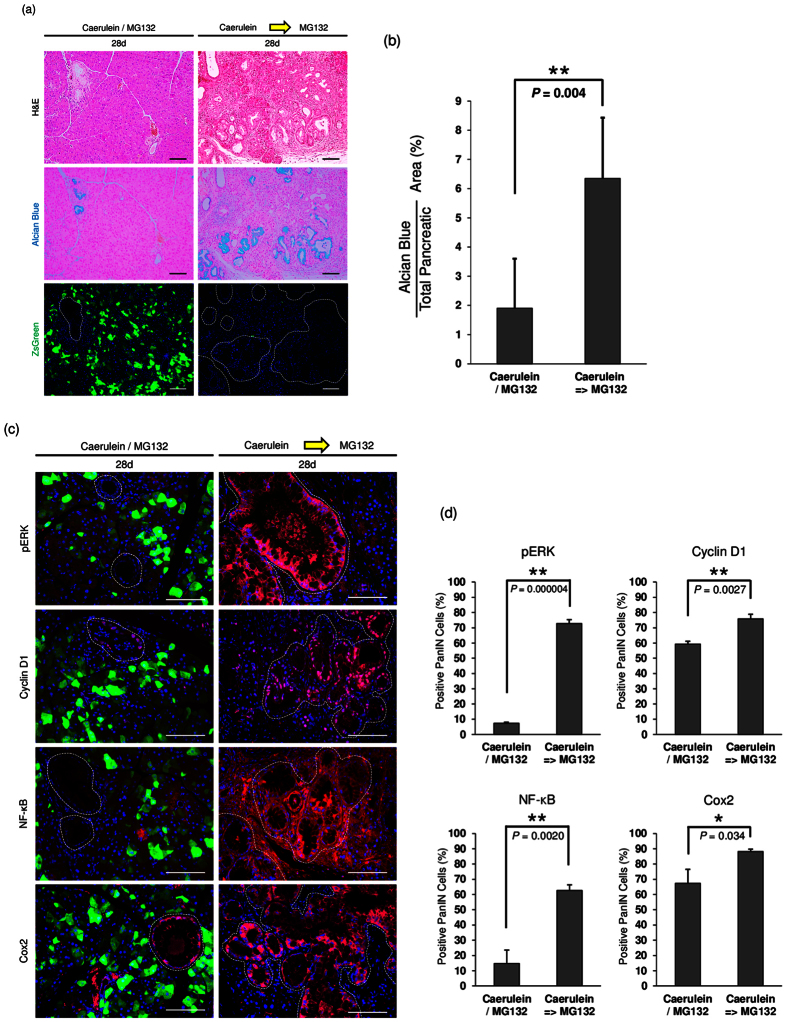
The effect of proteasome inhibitor after PanIN formation. *Gdeg;Pdx-1-Cre*;*LSL-Kras*^*G12D*^ mice were simultaneously treated with two sets of caerulein and MG132 on alternating days (left panels), or first treated with two sets of caerulein and after PanIN formation further treated with two sets of MG132 on day 14 and 16 (right panels). Mice were analyzed on day 28 (n = 3). (**a**) H&E staining, Alcian blue staining, fluorescent imaging of the pancreas of *Gdeg;Pdx-1-Cre*;*LSL-Kras*^*G12D*^ mice. (**b**) Quantification of Alcian blue positive areas at day 28. Values are shown as mean ± SD. (**c**) Immunofluorescence staining for pERK, cyclin D1, NF-κB and cox2 of the pancreas of *Gdeg;Pdx-1-Cre*;*LSL-Kras*^*G12D*^ mice. Dashed lines on the fluorescence images mark PanIN lesions. Bar, 100 μm. (**d**) Quantification of the positive staining rates of pERK, cyclin D1, NF-κB, and Cox2 in PanIN cells at day 28. Values are shown as mean ± SD.

We further assessed whether proteasome inhibition with MG132 affected PanIN lesions that had already formed. After development of PanIN, MG132 could not reduce PanIN lesions in the pancreas of *Gdeg;Pdx-1-Cre*;*LSL-Kras*^*G12D*^ mice (Fig. 6a), with the Alcian blue positive area of 6.35 ± 2.07% at day 28 (Fig. 6b). Immunofluorescence analysis showed that MG132 could not decrease the expression of pERK and its downstream cyclin D1, NF-κB, and Cox2 in the formed PanIN lesions accompanying increased proteasome activity (Fig. 6c). The positive staining rates of pERK, cyclin D1, NF-κB, and Cox2 in PanIN cells were 72.6% ± 2.6%, 75.8% ± 3.0%, 62.7% ± 3.6%, and 88.2% ± 1.5%, respectively (Fig. 6d). No significant difference was observed in the control mice with vehicle injection instead of MG132 (Supplementary Fig. 6). These observations indicated that proteasome activity might regulate the initiation of PanIN but not the maintenance of PanIN.

## Discussion

The proteasome is involved in a diverse array of biological processes with various tissue and/or cell-specific subtypes^29^,^30^. Cancer cells are largely dependent on the proteasome and proteasome activity is significantly activated in cancer cells with proliferating and hypermetabolic activities^9^,^11^. While the proteasome plays critical roles in advanced cancers including pancreatic cancer, its significance in the initiation of cancers are not disclosed. In the present study, we first established a transgenic mouse model with the Gdeg reporter. This Gdeg reporter system consists of ZsGreen fused to the degron motif^18^, whose accumulation indicates low proteasome activity. Expression of *Gdeg* mRNA in all examined pancreatic tissues was confirmed by RT-PCR analysis using LCM and FACS assays (Supplementary Fig. S2). The proteasome inhibitors induced the accumulation of Gdeg in primary cultured fibroblasts, indicating the transgene function in *Gdeg* mice (Supplementary Fig. S1).

**Figure 7:**
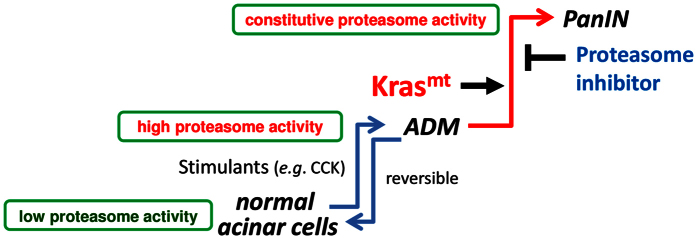
A proposed model for the role of proteasome activity in pancreatic carcinogenesis. Activation of proteasome activity plays crucial roles in *Kras*-induced PanIN formation. Stimulants such as caerulein induce briefly elevation of proteasome activity in pancreatic acinar cells and formation of ADM lesions, but both of them are transient in the absence of mutant KRAS. On the other hands, mutant KRAS causes constitutive elevation of proteasome activity and accelerates ADM to PanIN sequence.

ADM to PanIN sequence is the initial event of pancreatic carcinogenesis^5^,^6^. Though inflammatory insult to the pancreas transiently causes ADM, it is not sufficient to form PanIN and mutant KRAS is required for PanIN formation^7^,^8^. Understanding this process is critically important for developing early cancer detection methods and effective treatments. In combination with *Pdx-1-Cre;LSL-Kras*^*G12D*^ model, we successfully monitored proteasome activity in the initiation of PanIN. Our mouse model revealed that normal pancreatic acinar cells had low proteasome activity, whereas proteasome activity was definitely elevated in PanIN lesions. Caerulein stimulation induced briefly elevation of proteasome activity in pancreatic acinar cells and formation of ADM lesions, both of which were only transient in the absence of mutant KRAS. On the other hand, mutant KRAS caused constitutive elevation of proteasome activity and accelerated ADM to PanIN sequence, demonstrating the potential requirement of proteasome activity in *Kras*-induced PanIN formation (Fig. 7). The significance of proteasome activity was further verified by the proteasome inhibitor, which clearly reduced PanIN formation without affecting ADM events. It was noteworthy that the proteasome inhibitor had no effect on PanIN lesions that had already formed, indicating that ADM to PanIN sequence is irreversible and proteasome activity is especially required for the initiation of PanIN. Although Gdeg reduction was improved by proteasome inhibitor without affecting pancreatic damage (Supplementary Fig. 3), we cannot rule out the effects of factors other than proteasome activity such as stress-induced mRNA and lysosomal degradation on Gdeg reduction, and this is one of the limitations of our study.

To further understand the effects of proteasome inhibition, we focused on the *RAS*-induced oncogenic pathways. The RAS/MAPK/ERK pathway is activated in many cancers and participates in the regulation of a large variety of processes^31^,^32^. In our study, the RAS/MAPK/ERK pathway was active in PanIN lesions, as confirmed by strong immunostaining of pERK. In contrast, decreased levels of pERK were observed when proteasome activity was inhibited. ERK activity regulates the induction of cyclin D1^23^. Overexpression of cyclin D1 shortens the G_1_-S transition and promotes cell progression and differentiation. The NF-κB pathway is another important pathway that is crucial in mediating inflammation-induced tumor growth and progression^24^,^33^. Cox2 is regulated by NF-κB and promotes cell survival, proliferation, and angiogenesis and inhibit apoptosis^25^. All of these factors were activated in PanIN lesions, and were reduced by proteasome inhibition in this study. Although these data cannot exclude the consequence of PanIN reduction after proteasome inhibition, it may be possible that proteasome activity is involved in pERK levels and its downstream pathways associated with cell cycle and inflammation. A limitation of our study is the lack of molecular evidence, and the mechanistic insights into the effect of proteasome inhibitors including MG132 and other compounds such as borezomib should be further investigated.

In summary, elevation of proteasome activity plays a critical role in the initiation of PanIN. Proteasome activity is worthy of further study as a key component of pancreatic carcinogenesis.

## Methods

### Transgenic mouse lines

The degron sequence of the ornithine decarboxylase (ODC) is known to be directly recognized by proteasomes^18^, which leads to the immediate destruction of the involved protein. The ZsGreen-degron^ODC^ (Gdeg) reporter expresses the green fluorescent protein ZsGreen fused to the degron of ODC, which intracellularly accumulates as a result of the low activity of the 26S proteasome. We constructed a 2.7-kb transgene including the Gdeg reporter combined with the cytomegalovirus (CMV) promoter and the simian virus 40 (SV40) poly-adenylation site (Fig. 1a). The purified transgene was microinjected into fertilized oocytes derived from ICR mice at the single-cell stage. The transgene was identified by polymerase chain reaction (PCR) analysis following amplification of a unique 1.6-kb fragment using the following primers specific for the CMV promoter: 5′-GTCATTAGTTCATAGCCCATATATGGAGTT-3′ and the SV40 sequencesx: 5′-GCAGTGAAAAAAATGCTTTATTTG-3′.

*Gdeg* transgenic mice were further crossed with *Pdx-1-*Cre; *LSL-Kras*^*G12D*^ mice^5^ (provided by Dr. David Tuvenson, Cold Spring Harbor Laboratory, Long Island, NY) to create *Gdeg; Pdx-1-Cre; LSL-Kras*^*G12D*^ triple transgenic mice.

### Caerulein and MG132 treatment

For caerulein treatment, 8-week old *Gdeg* mice or *Gdeg; Pdx-1-Cre*; *LSL-Kras*^*G12D*^ mice were intraperitoneally injected with caerulein (50 μg/kg; Sigma-Aldrich) or PBS on 2 alternating days once every hour for 6 hours each day^7^. The final day of caerulein injection was considered day 0.

For MG132 treatment, MG132 (Calbiochem, San Diego, CA) was dissolved in dimethyl sulfoxide (DMSO) and intraperitoneally administered. At 8 weeks of age, *Gdeg* mice or *Gdeg; Pdx-1-Cre*; *LSL-Kras*^*G12D*^ mice were administered with MG132 at a dose of 5 mg/kg dissolved in 50 μl of DMSO or with vehicle alone together with 6 hourly injections of caerulein (50 μg/kg). The series of MG132 and caerulein injections were repeated on alternating days. After two sets of MG132 and caerulein injections, mice were analyzed on day 14 or 28.

For MG132 treatment after PanIN formation, 8-week old *Gdeg; Pdx-1-Cre*; *LSL-Kras*^*G12D*^ mice were first treated with 6 hourly injections of caerulein (50 μg/kg) on two alternating days. Mice were further treated with MG132 at a dose of 5 mg/kg dissolved in 50 μl DMSO on day 14 and 16, and analyzed on day 28.

Protocols were approved by the Animal Care Committee of Tokyo Medical and Dental University (Permission No. 0150044), and all mouse experiments were carried out in accordance with the approved guidelines.

### Immunofluorescence and immunohistochemistry

Pancreatic tissues were fixed overnight in 4% paraformaldehyde as previously described^16^, embedded in paraffin, and sectioned (3.5 μm in thickness). Hematoxylin and eosin (H&E) staining and Alcian blue staining were performed. For immunofluorescence, tissue sections were prepared according to standard procedures. After deparaffinization and antigen retrieval in 10 mM sodium citrate buffer pH 6.0, slides were incubated in permeabilization buffer (0.1% Triton X-PBS) for 30 minutes, followed by incubation in blocking buffer (3% bovine serum albumin-PBS) for 30 minutes and subsequent exposure to the primary antibodies overnight at 4 °C. The primary antibodies used in this study are listed in supplementary table 1. The sections were then treated for 1 hour with the secondary antibody and Hoechst 33342 solution for nuclear staining. Alexa Fluor 568 donkey anti-rabbit, Alexa Fluor 647 goat anti-rabbit, Alexa Fluor 647 goat anti-mouse, and Alexa Fluor 647 chicken anti-goat antibodies (Molecular Probes; Invitrogen) were used as the secondary antibodies at a dilution of 1:500. After mounting, the slides were visualized with a fluorescence microscope (Carl Zeiss, Oberkochen, Germany).

### Evaluation of Alcian blue positive areas

To quantify relative Alcian blue positive area, six random, non-overlapping images were obtained at a magnification of x200. For each image, the Alcian blue positive area and the total pancreatic epithelial area were measured using cellSens Dimension software version 1.6 (Olympus, Tokyo, Japan), and the percentage of the Alcian blue positive area was calculated.

### Statistical analysis

Data are presented as mean ± standard deviation (SD). *P*-values were acquired with the Student’s *t* test using SPSS version 21.0 (SPSS, Chicago, IL). *P* < 0.05 was considered statistically significant.

## Additional Information

**How to cite this article**: Furuyama, T. *et al.* Proteasome activity is required for the initiation of precancerous pancreatic lesions. *Sci. Rep.* **6**, 27044; doi: 10.1038/srep27044 (2016).

## Supplementary Material

Supplementary Information
