# Supplementary material for: Proteasome activity is required for the initiation of precancerous pancreatic lesions
Source: Sci Rep. 2016 May 31;6:27044. doi: 10.1038/srep27044 (PMC4886684; doi:10.1038/srep27044)
Supplement: Supplementary Information [file srep27044-s1.pdf]

Proteasome activity is required for the initiation of precancerous pancreatic lesions

Takaki Furuyama[1,2], Shinji Tanaka[1,2*], Shu Shimada[1], Yoshimitsu Akiyama[1], Satoshi Matsumura[2], Yusuke Mitsunori[2], Arihiro Aihara[2], Daisuke Ban[2], Takanori Ochiai[2], Atsushi Kudo[2], Hiroshi Fukamachi[1], Shigeki Arii[2], Yoshiya Kawaguchi[3], Minoru Tanabe[2]

Supplementary Information

Supplementary Methods

Primary culture of fibroblasts

For primary culture of fibroblasts, the lungs harvested from *Gdeg* transgenic mice were cut into small pieces and placed in Dulbecco's modified Eagle's medium (DMEM; Invitrogen, Carlsbad, CA). Migrated fibroblasts were further cultured in DMEM supplemented with 10% fetal bovine serum (Sigma-Aldrich, St. Louis, MO) and penicillin/streptomycin (Sigma-Aldrich) as antibiotics. To confirm stable gene transfection, primary cultured fibroblasts were exposed to the proteasome inhibitor MG132 (Calbiochem, San Diego, CA) for 24 hours at a concentration of 10μM or bortezomib (AdooQ Bio Science, Irvine, CA) for 9 hours at a concentration of 100nM.

Laser capture microdissection (LCM)

Formalin-fixed paraffin-embedded (FFPE) samples were sectioned at 10 μm and mounted on MembraneSlide 1.0 PEN (Carl Zeiss, Jena, Germany). FFPE sections were deparaffinized by a series of xylene and ethanol, and then stained with toluidine blue. Once air-dried, the regions of islet, acinar and PanIN cells were laser microdissected with a PALMRMicroBeam laser system (Carl Zeiss).

Fluorescence activated cell sorting (FACS)

Isolation of pancreatic cells of the *Gdeg* mice was performed as previously described [1]. Briefly, after sacrificing the *Gdeg* mice, pancreatic tissues were minced with scissors, and exposed to Collagenase P (Roche) for 15 minutes at 37 °C. Isolated pancreatic cells were washed in Hank's balanced salt solution containing fetal bovine serum, filtered through nylon mesh (BD Falcon), and then sorted using FACS Aria II (BD Biosciences).

RT-PCR analysis

Total RNA was extracted by using a NucleoSpin® totalRNA FFPE XS (TAKARA BIO INC., Shiga, Japan) according to the manufacturer's instructions. cDNA was synthesized using random hexamers as primers and a SuperScript III Reverse Transcriptase (Thermo Fisher Scientific Inc., Waltham, MA). First step RT-PCR was conducted with 32 or 35 cycles. PCR products were diluted at 1:100 and then used for nested RT-PCR with 32 cycles. Each PCR cycle consisted of 95ºC for 30 sec, 58ºC for 30 sec and 72ºC for 30 sec, followed by a final extension at 72˚C for 5 min. The PCR products were electrophoresed in 3% agarose gels containing 0.5µg/mL ethidium bromide. Primer sequences and the reaction conditions are shown in Supplementary Table 2.

Supplemenary Reference

1 Shi, G. et al. Maintenance of acinar cell organization is critical to preventing Kras-induced acinar-ductal metaplasia. *Oncogene* **32**, 1950-1958 (2013).

Supplementary Figure 1

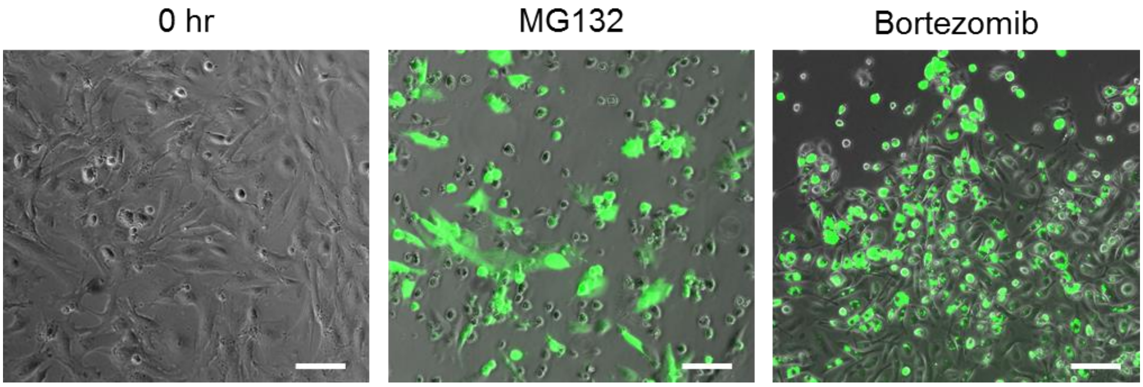

Supplementary Figure 1 | Primary cultured fibroblasts from *Gdeg* mice.

Fluorescence imaging of primary cultured fibroblasts from *Gdeg* mice were treated with MG132 at a concentration of 10μM or bortezomib at a concentration of 100nM.

Supplementary Figure 2

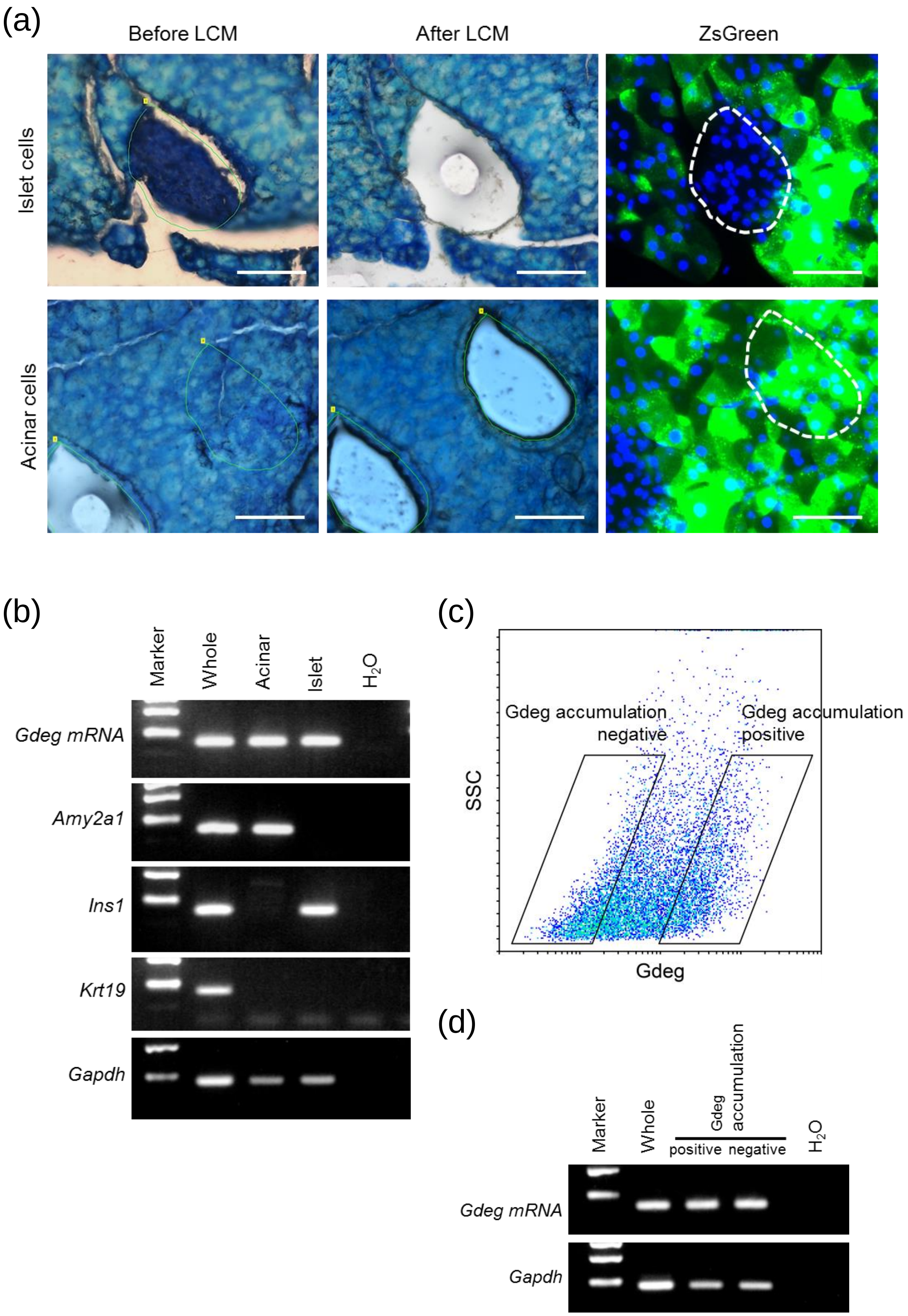

Supplementary Figure 2 | RT-PCR analysis of pancreatic cells from *Gdeg* mice. (a) Laser capture microdissection of pancreatic tissues from *Gdeg* mice. (b) RT-PCR analysis of *Gdeg* mRNA expression in laser microdissected tissues. RNA from a whole pancreatic tissue is used as a positive control. The regions dissected from acinar and islet cells specifically expressed *Amyl2a2* (amylase) and *Ins1* (insulin), respectively. Expression of *Gapdh* certified the quality of mRNA in each sample. *Krt19, keratin 19.* Marker: 100 bp ladder. (c) Isolation of the Gdeg accumulation-negative and Gdeg accumulation-positive pancreatic cells using FACS. (d) RT-PCR analysis of *Gdeg* mRNA expression in pancreatic cells after FACS. RNA from a whole pancreatic tissue is used as a positive control. Expression of *Gapdh* certified the quality of mRNA in each sample. Marker: 100 bp ladder.

Supplementary Figure 3

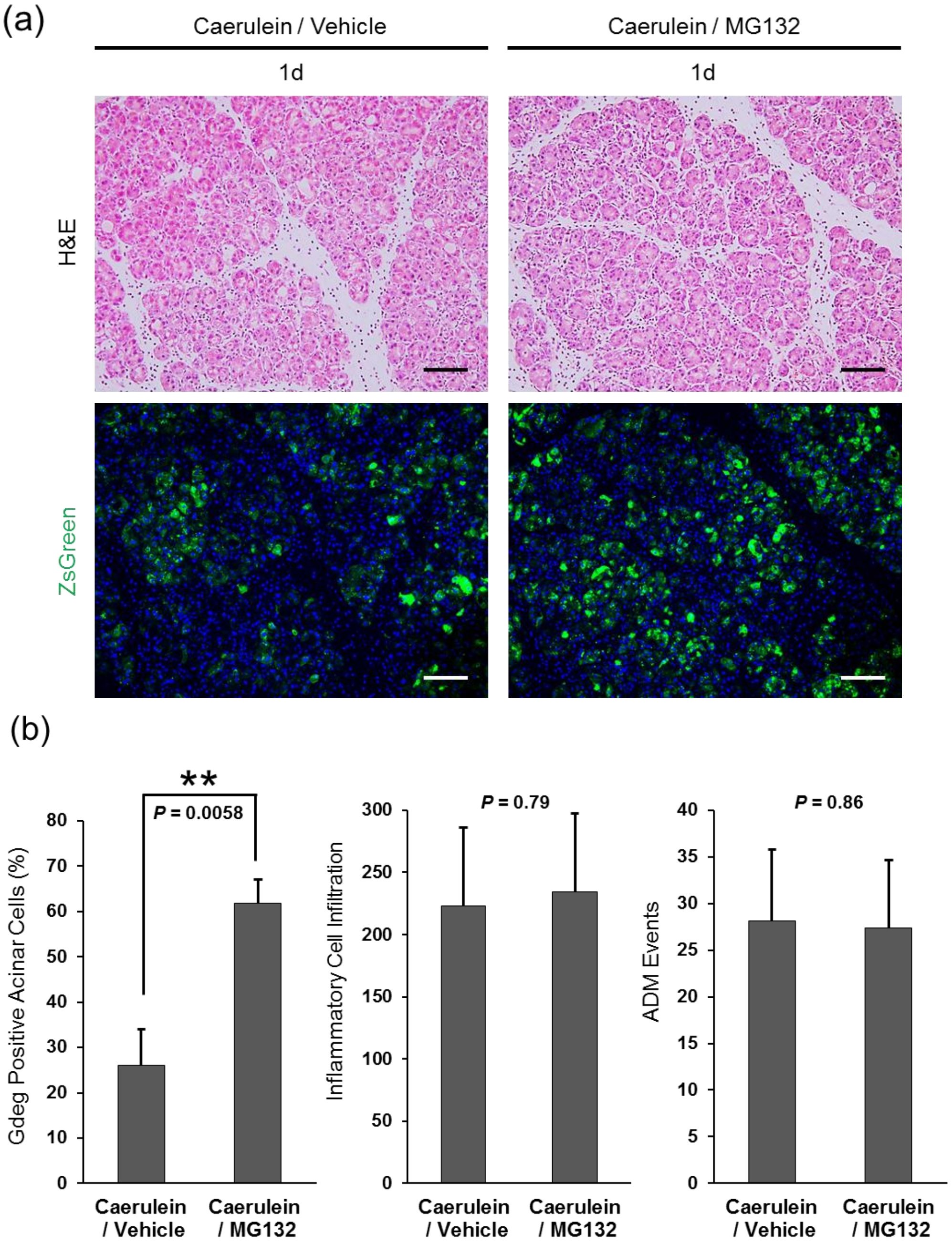

Supplementary Figure 3 | Pancreatic tissues of *Gdeg* mice at day1 after caerulein and MG132 treatment.

(a) H&E staining and fluorescence imaging of the pancreas of caerulein-treated *Gdeg* mice with or without MG132 administration (n = 4). Bar, 100 µm.

(b) Quantification of Gdeg-Positive acinar cells, inflammatory cell infiltration, and ADM events. Values are shown as mean ± SD.

Supplementary Figure 4

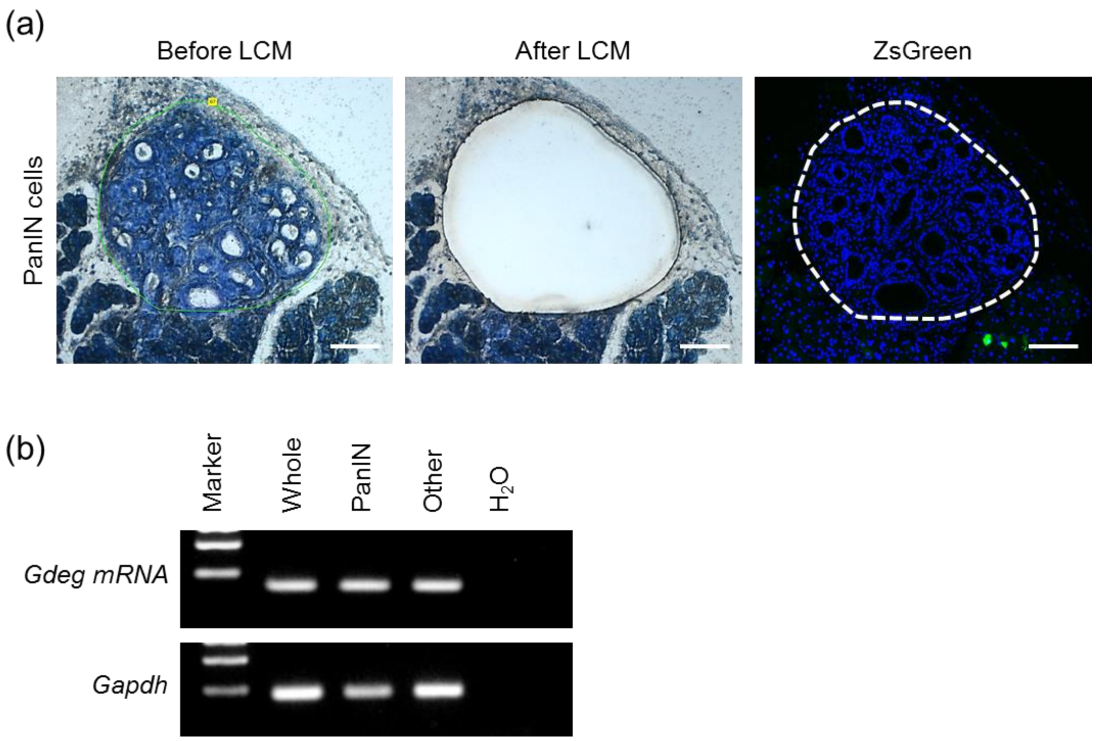

Supplementary Figure 4 | RT-PCR analysis of PanIN cells from *Gdeg* mice.

(a) Laser capture microdissection of PanIN cells. (b) RT-PCR analysis of *Gdeg* mRNA expression in PanIN cells. Other indicated outside PanIN region containing acinar and islet cells. RNA from a whole pancreatic tissue is used as a positive control. Expression of *Gapdh* certified the quality of mRNA in each sample. Marker: 100 bp ladder.

Supplementary Figure 5

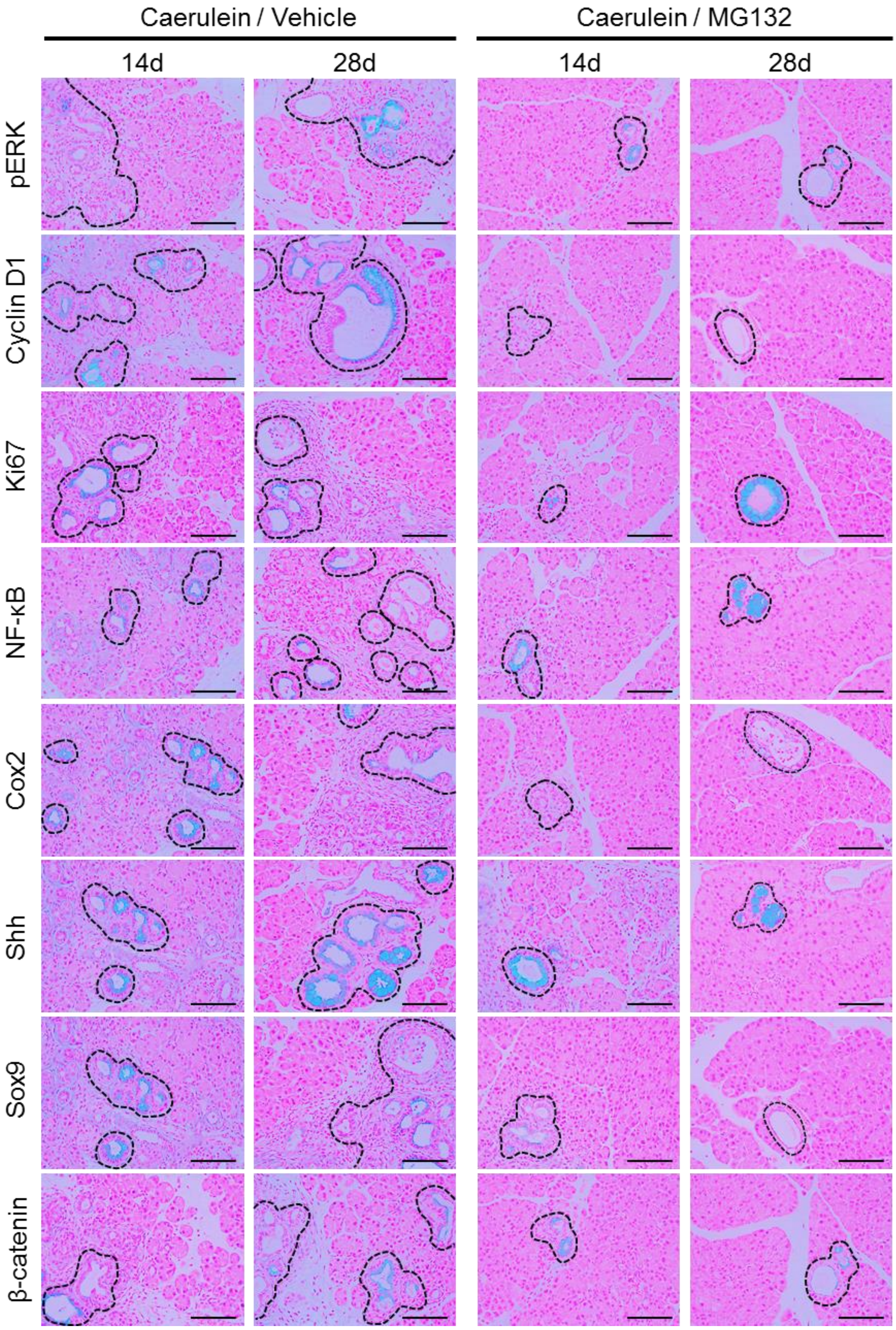

Supplementary Figure 5 | Alcian blue staining corresponding to immunofluorescence staining in Figure 5.

Dashed lines on the images mark PanIN lesions. Bar, 100 μm.

Supplementary Figure 6

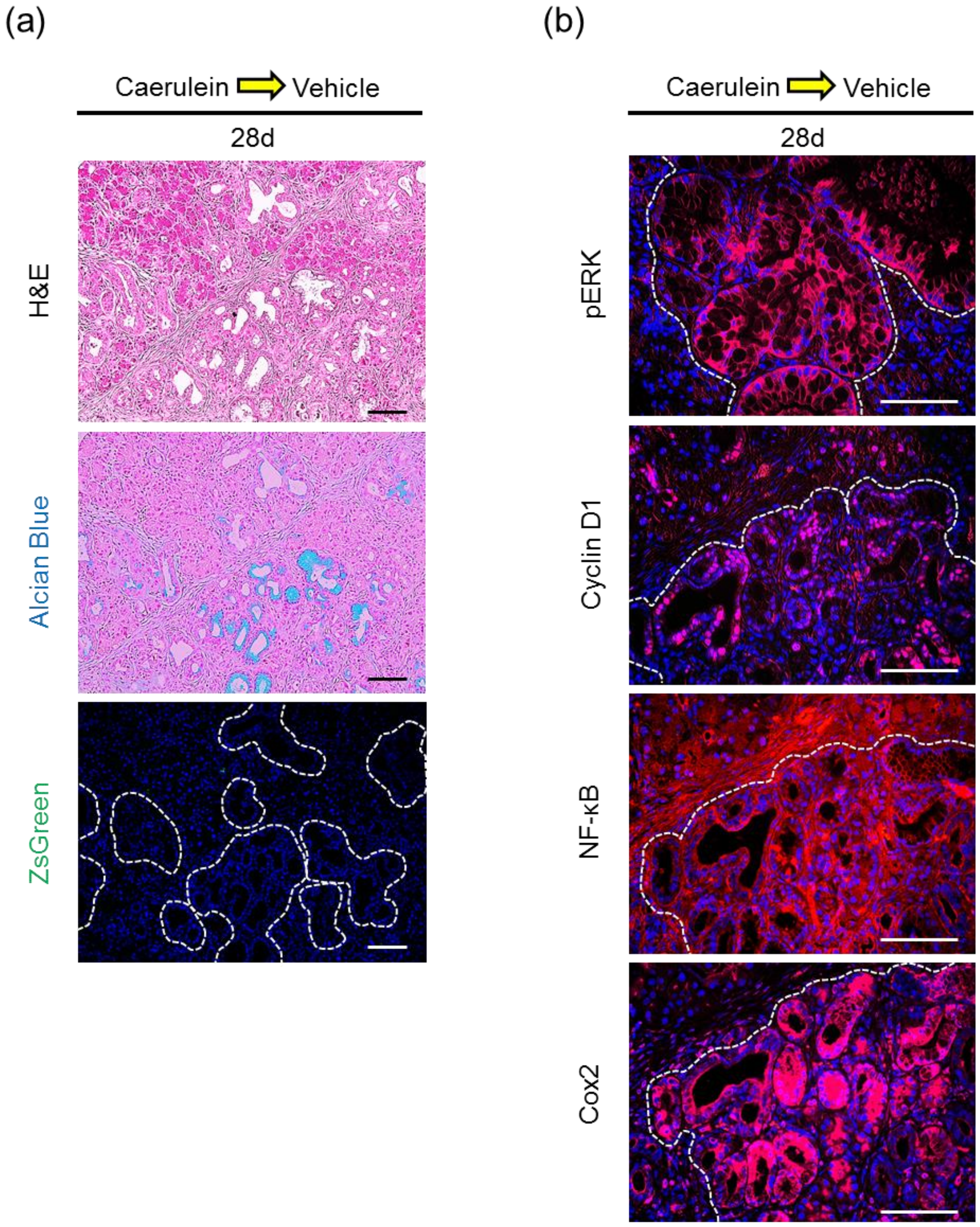

Supplementary Figure 6| Control mice with vehicle injection after PanIN formation.

Gdeg;Pdx-1-Cre;*LSL-Kras*^{G12D} mice were first treated with two sets of caerulein and after PanIN formation further treated with two sets of vehicle on day 14 and 16. Mice were analyzed on day 28 (n=3).

(a) H&E staining, Alcian blue staining, fluorescent imaging of the pancreas. The Alcian blue positive area was 6.39 ± 2.45%.

(b) Immunofluorescence staining for pERK, cyclin D1, NF-κB and cox2 of the pancreas of *Gdeg;Pdx-1-Cre*;*LSL-Kras*^{G12D} mice. Dashed lines on the fluorescence images mark PanIN lesions. Bar, 100 µm. The positive staining rates of pERK, cyclin D1, NF-κB, and Cox2 in PanIN cells were 71.2 ± 2.24%, 73.7 ± 4.3%, 59.0 ± 1.2%, and 91.7 ± 1.7%, respectively.

Supplementary Table 1 Antibodies.

Antibody	Supplier	Catalog Number	IF Dilution	IHC Dilution
Amylase	Sigma-Aldrich	A8273	1:500	-
Cytokeratin19	Dako	M088801	1:50	-
pERK (phospho-p44/42)	Cell Signaling	4370	1:50	1:50
Cyclin D1	Abcam	ab16663	1:100	1:100
Ki67	Abcam	ab16667	1:100	-
NF-κB (p65)	Cell Signaling	8242	1:200	1:200
Cox2	Abcam	ab15191	1:200	1:200
αSMA	Abcam	ab5694	1:200	-
Shh	R&D Systems	AF445	1:100	-
Sox9	Millipore	AB5535	1:500	-
β-catenin	Santa Cruz Biotechnoogy	sc-7199	1:50	-

Supplementary Table 2 Primer sequences and their PCR conditions in this study.

Type of PCR [1]	Name	Sense	Antisense	Size of the PCR products (bp)	Cycles	Symbol (GenBank Accession No.)
1st PCR	ZsGreen-degronODC	CATCACCGTGAGCGTGGAGGA	CCAGTTGTCGGTCATCTTCTTCAT	106	32	
	Amylase 2a1	TGGGAAAGATACCAACCAATCAGC	GACAGCATCCACATAAATACGGAC	118	35	Amy2a1 (XM_011240373)
	insulin I	CCCAGCCCTTAGTGACCAGCTA	AGAGGGCAAGCAGGGCCAGCA	109	35	Ins1 (NM_008386)
	Keratin 19	CCTCCCGAGATTACAACCACT	TCATCTGCAGCCAGGCGAGCAT	124	35	Krt19 (NM_008471)
	Gapdh	GCCAAGGTCATCCATGACAACTT	AGGGATGATGTTCTGGGCAGC	144	32	Gapdh (NM_001289726)
Nested PCR	ZsGreen-degronODC	GAACTGCATGTACCACGAGTC	CTTCTTCATCACGGGGCCGTC	70	32	
	Amylase	AAATCTGCACAAGGTCTGGAAATG	CGGACACCAACATTGTTGCACC	73	32	
	insulin I	TAATCAGAGACCATCAGCAAGCAG	AGCAGGGGTAGGAAGTGCACCA	70	32	
	Keratin 19	ACTTTAAGACCATCGAGGACTTGC	TGTCAATCTGTAGGACAATCTTGGA	81	32	
	Gapdh	GTGGAAGGGCTCATGACCACAG	CGGCCATCACGCCACAGCTTTC	89	32	

1) Amplicons of 1st PCR were diluted at 1:100, and then used for nested PCR. Annealing temperature of 1st and nested PCR was 58°C.
